# Supplementary material for: Wobble tRNA modification and hydrophilic amino acid patterns dictate protein fate
Source: Nat Commun. 2021 Apr 15;12:2170. doi: 10.1038/s41467-021-22254-5 (PMC8050329; doi:10.1038/s41467-021-22254-5)
Supplement: Supplementary file 1 — Supplementary Information [file 41467_2021_22254_MOESM1_ESM.pdf]

SUPPLEMENTARY FIGURE LEGENDS

Supplementary Figure 1. Analysis of U_{34}-codons in the human orfeome.

a, Basal luminescence of MDA-MB231 cells stably expressing the indicated constructs (n=6 except for AAA where n=4 independent experiments, data are mean + s.d). **b, c, d,** Levels of indicated proteins in MDA-MB231 cells stably overexpressing the AAG-AAA (b), the CAG-CAA (c) or the GAG-GAA (d) constructs, infected with IPTG-inducible (48h) shRNA constructs for ELP3 and CTU2, were determined by western blot (n=2 replicates). **e, f**, NanoLuc/Firefly luminescence ratio (e, n=3 independent experiments) and protein levels (f, n=2 replicates) of MDA-MB231 cells stably expressing a WT NanoLuc/Firefly construct upon IPTG-inducible (48h) depletion of ELP3 and CTU2 (two-sided *t*-test, data are mean + s.d). **g, h**, Genes enriched (q value <0.05) in either one of the U_{34}-codons (g) or in all three of them (h; Venn diagram). **i, j**, Bubble plot for the GO-term analysis (no pathway=no consensus pathway) (i) and table for gene family enrichment analysis (j) of the U_{34}-enriched genes (n=608, AAA&CAA&GAA, q<0.05). **k, l**, Protein levels (k, n=1 replicate) and mRNA levels (l) of indicated genes were assessed by western blot and qRT-PCR (n=2 independent experiments) in MDA-MB231 cells overexpressing a FLAG-RPL22 construct upon depletion or not of ELP3 or CTU2; data are mean + s.d.

Supplementary Figure 2. Protein fate upon depletion of the U_{34}-enzymes is poorly predicted by mRNA codon content.

a, b, Volcano plot of the proteomics (n= 3 independent experiments, a) and RNA-seq (n= 3 independent experiments, b) of BT549 cells depleted or not of ELP3**. c,** Frequency of U_{34}-codons (q value<0.05) and protein expression of proteins in BT549 cells depleted of ELP3 was plotted, correlation was calculated by Spearman test (two-sided). **d, e, f,** qRT-PCR (n=2

independent experiments) for the indicated genes of MDA-MB231 (d) and BT549 (e) cells depleted of ELP3 or CTU2; and of MDA-MB231 cells overexpressing a FLAG-RPL22 construct upon depletion or not of ELP3 or CTU2 (f).

Supplementary Figure 3. U_{34}-target proteins are directed towards aggregates upon U_{34}-enzymes depletion.

a, MCF7 cells depleted or not of ELP3 and CTU2, were treated for 24h with 5μM of MG132 or for 6h with 100μM chloroquine as indicated. Protein levels were detected by western blot (n=1 replicate). **b**, qRT-PCR of MCF7 cells overexpressing a FLAG-RPL22 construct and KIF4A-WT or -Mut constructs upon depletion or not of ELP3 or CTU2 (n=2 independent experiments). **c**, Protein aggregation was measured by FACS in MCF7 cells overexpressing KIF4A-WT or KIF4A-Mut upon depletion of ELP3 or CTU2 (n=2 independent experiments, data are mean +sd).

Supplementary Figure 4. Identification of mRNA features clustering proteins whose fate depends on the presence of U_{34}-enzymes.

a, Schematic representation of the performed feature discovery analysis. **b**, mRNA expression (TGCA) of the U_{34}-enriched genes downregulated or not upon ELP3 depletion in patients' dataset. One way-ANOVA. Data show median of expression. **c**, mRNA length of the U_{34}-enriched genes downregulated (n=58) or not (n=56) upon ELP3 depletion was assessed (two-ways t-test); data are mean +/- SEM. **d**, Principal component analysis of the codon content of the U_{34}-enriched genes downregulated or not upon ELP3 depletion ($\chi 2$ test, two-sided). **e**, Distribution of AAA, CAA, and GAA codons along the proteins of the U_{34}-enriched genes downregulated or not upon ELP3 depletion (one way ANOVA, data are mean).

Supplementary Figure 5. Protein and aggregation features to predict protein fate upon depletion of U_{34}-enzymes.

a, b, Principal component analyses of the number of secondary structures (a) and amino acid content (b) of the U_{34}-enriched genes downregulated or not upon ELP3 depletion ($\chi 2$ test, two-sided; n.s.: non-significant). **c-f,** Aggregation propensity (zyggregator, c), total charge (d), hydrophobicity (e) and hydrophobic pentasequences (f) of the U_{34}-enriched genes downregulated (n=58) or not (n=56) upon ELP3 depletion was assessed (two-ways t-test; n.s.: non-significant); data are mean +/- SEM.

Supplementary Figure 6. Identification of the consensus hydrophilic motifs.

a, Distribution of the hydrophilic motif orfeome-wide. **b**, Distribution of the hydrophilic motif across protein length (Kolmogorov-Smirnov test, two-sided). **c**, Correlation between the number of hydrophilic motifs and protein length (Pearson correlation, two-sided). **d, e,** Conservation score of hydrophilic motifs (d; n=2478) and random sequences of 5 amino acids (e; n=10.000) across 10 mammalian species.

Supplementary Figure 7. The hydrophilic motif predicts the aggregation of U_{34}-target proteins upon U_{34}-enzymes depletion.

a, b, qRT-PCR after ribosome immunoprecipitation (left) or total RNA (right) of FLAG-RPL22 overexpressing MCF7 cells expressing KIF4A mutants (a) or KIF5B mutants (b) upon ELP3 depletion (*n* = 2 independent experiments, data are mean + s.d.).

Supplementary Figure 8. The hydrophilic motif is not associated with known aggregation /degradation pathways.

a, Global propensity aggregation (beta-aggregation) of KIF4A-WT and KIF4A-Del assessed

by the TANGO algorithm (two-sided *t*-test). **b,** Dot plot of aggregation propensity (logK) and number of hydrophilic pentamers in proteins enriched in U_{34}-codons (q<0.05) downregulated or not upon U_{34}-enzymes depletion (Spearman correlation, two-sided). **c,** Schematic representation of proteins whose expression depends on the U_{34}-enzymes. Pink boxes represent hydrophilic pentamers. **d, e,** Dot plot of CMA motifs and number of hydrophilic pentamers in proteins enriched in U_{34}-codons (q<0.05) downregulated (e) or not (d) upon U_{34}-enzymes depletion (Spearman correlation, two-sided).

SUPPLEMENTARY DATA.

Supplementary Data 1: Gene set enrichment analysis of U_{34}-codons enriched human orfeome. Bubble chart of the GSEA analysis of AAA&CAA&GAA q value <0.05 genes.

Supplementary Data 2: U_{34}-codons enrichment analysis. Calculation of U_{34}-codons enrichment statistical significance by chi-square test (two-sided) in proteomics and RNA-seq of BT549 depleted of ELP3; and in the kinesin family.

Supplementary Data 3: U_{34}-enzymes protein targets, U_{34}-codon composition and presence of hydrophilic pentasequence. List of proteins down regulated upon depletion of ELP3, their content in U_{34}-codons and the presence of hydrophilic pentasequence.

Supplementary Data 4: Hydrophilic and U_{34}-codons enrichment as key characteristic of U_{34}-target proteins. Calculation of U_{34}-codons enrichment & motif presence statistical significance by chi-square test of downregulated proteins and aggregates in BT549 depleted of ELP3.

Supplementary Data 5: List of materials.

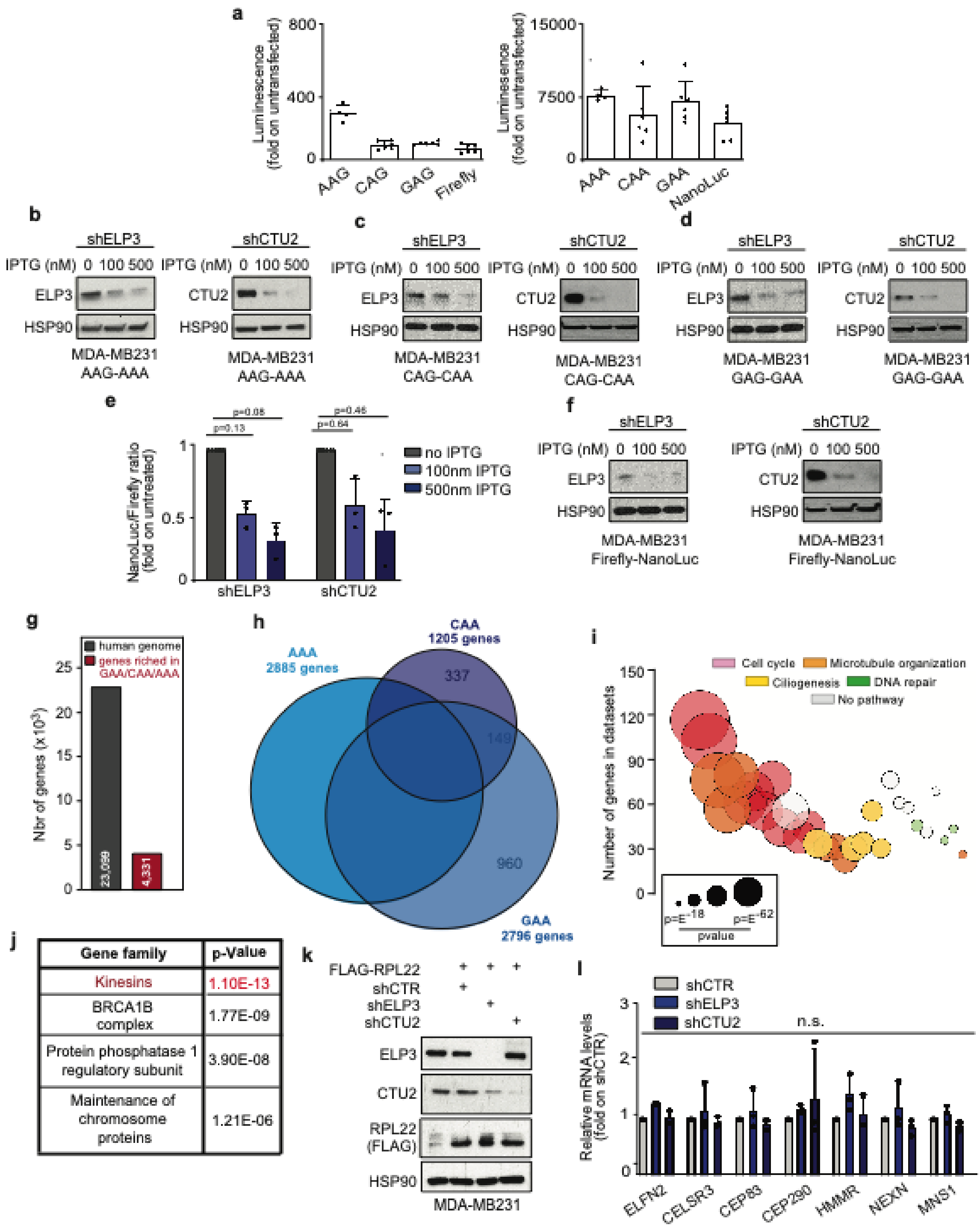

Gene family	p-Value
Kinesins	1.10E-13
BRCA1B complex	1.77E-09
Protein phosphatase 1 regulatory subunit	3.90E-08
Maintenance of chromosome proteins	1.21E-06

Supplementary Figure 1 related to Figure 1

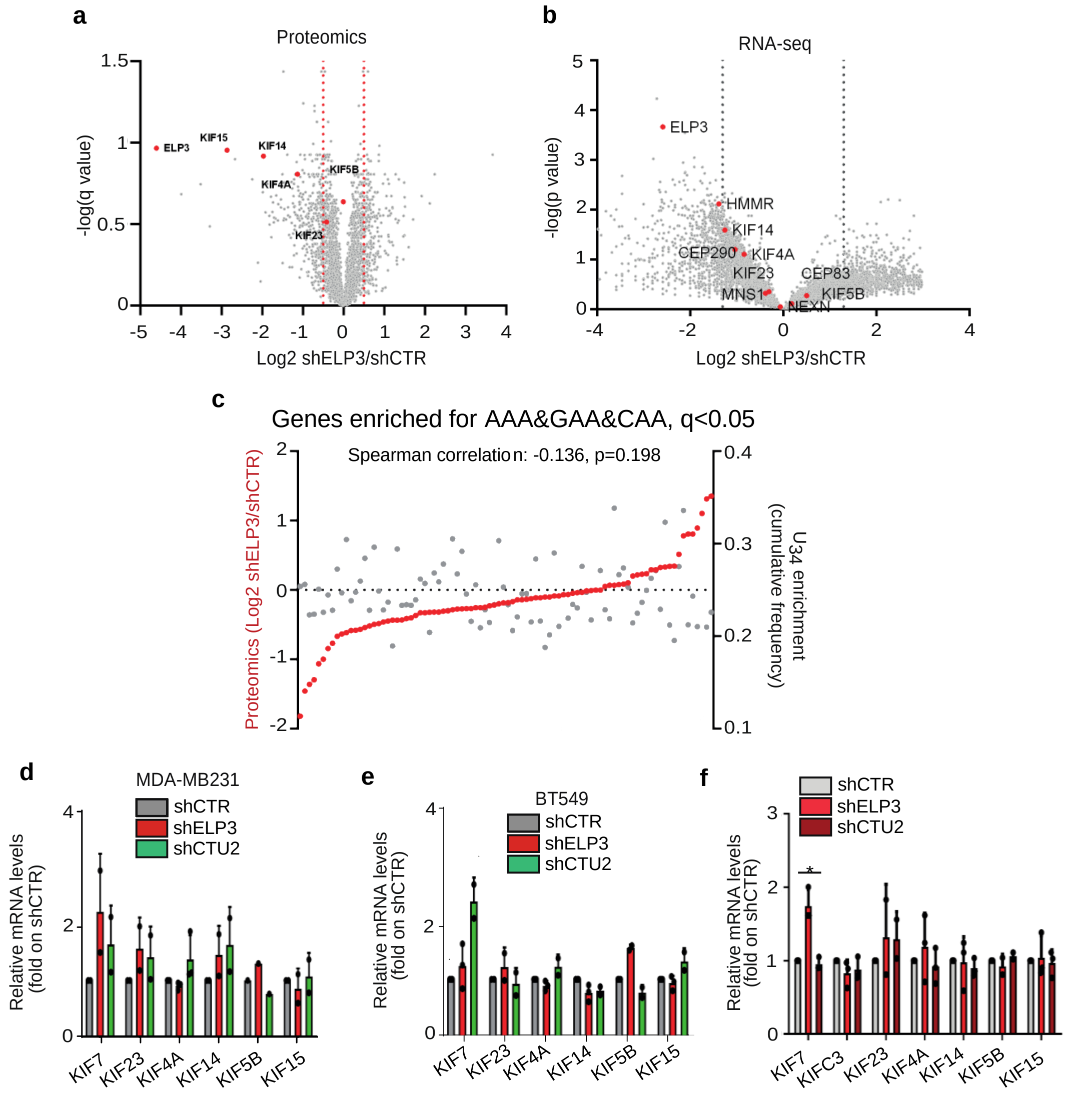

Supplementary Figure 2 related to Figure 2

99

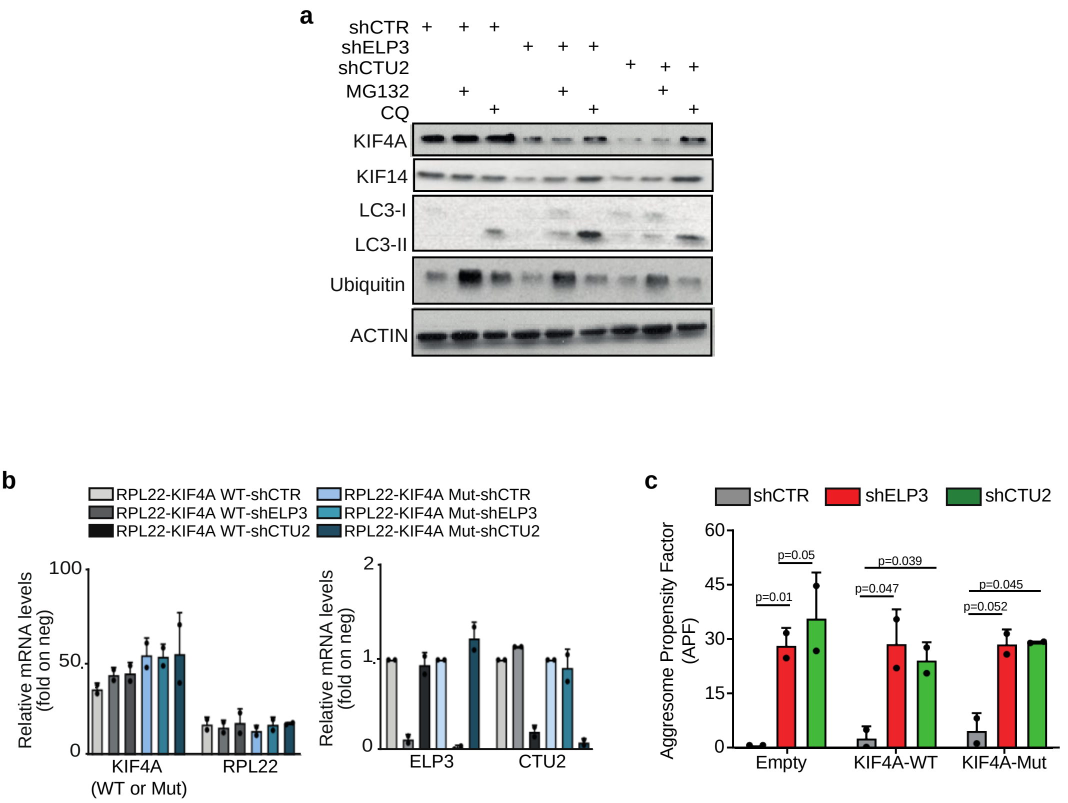

Supplementary Figure 3 related to Figure 3

100

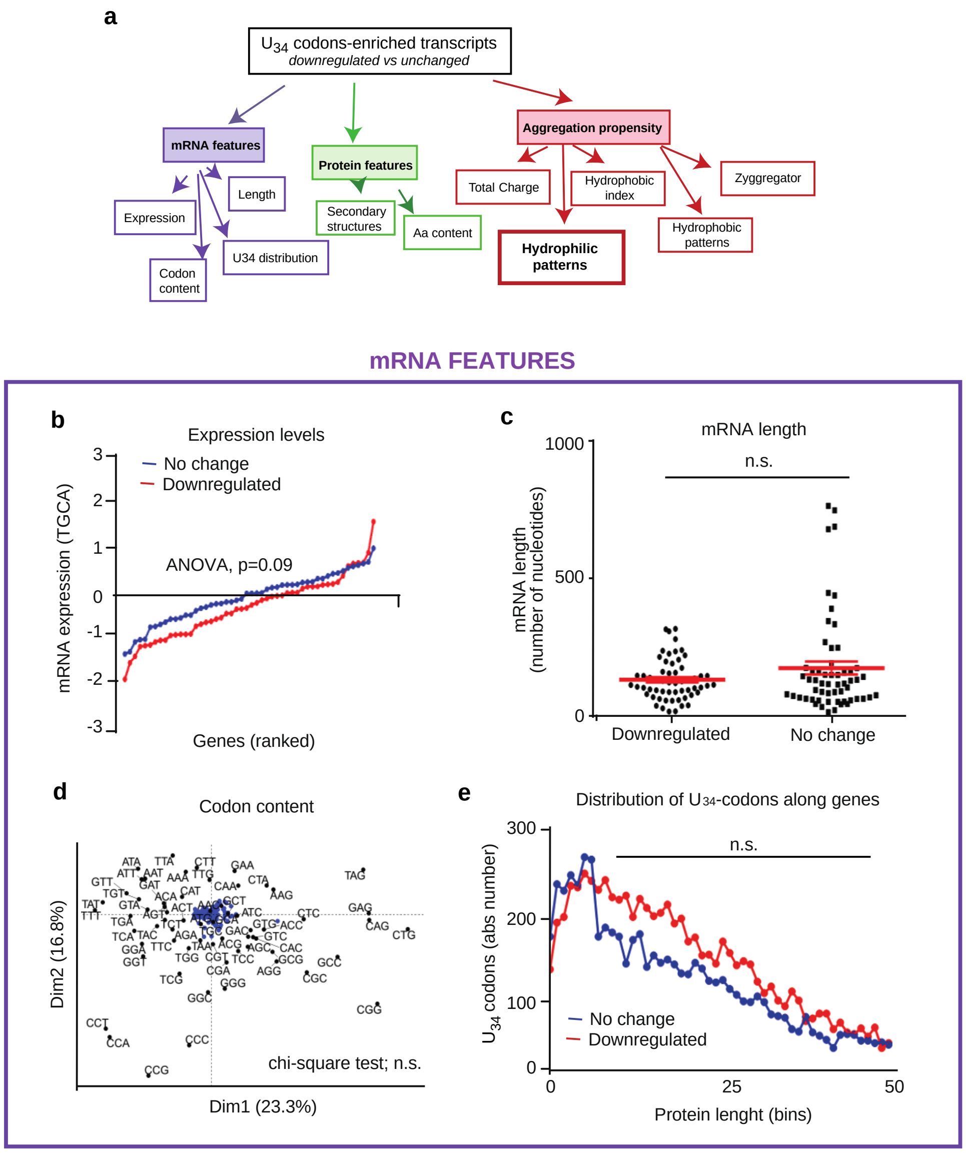

Supplementary Figure 4 related to Figure 4

101

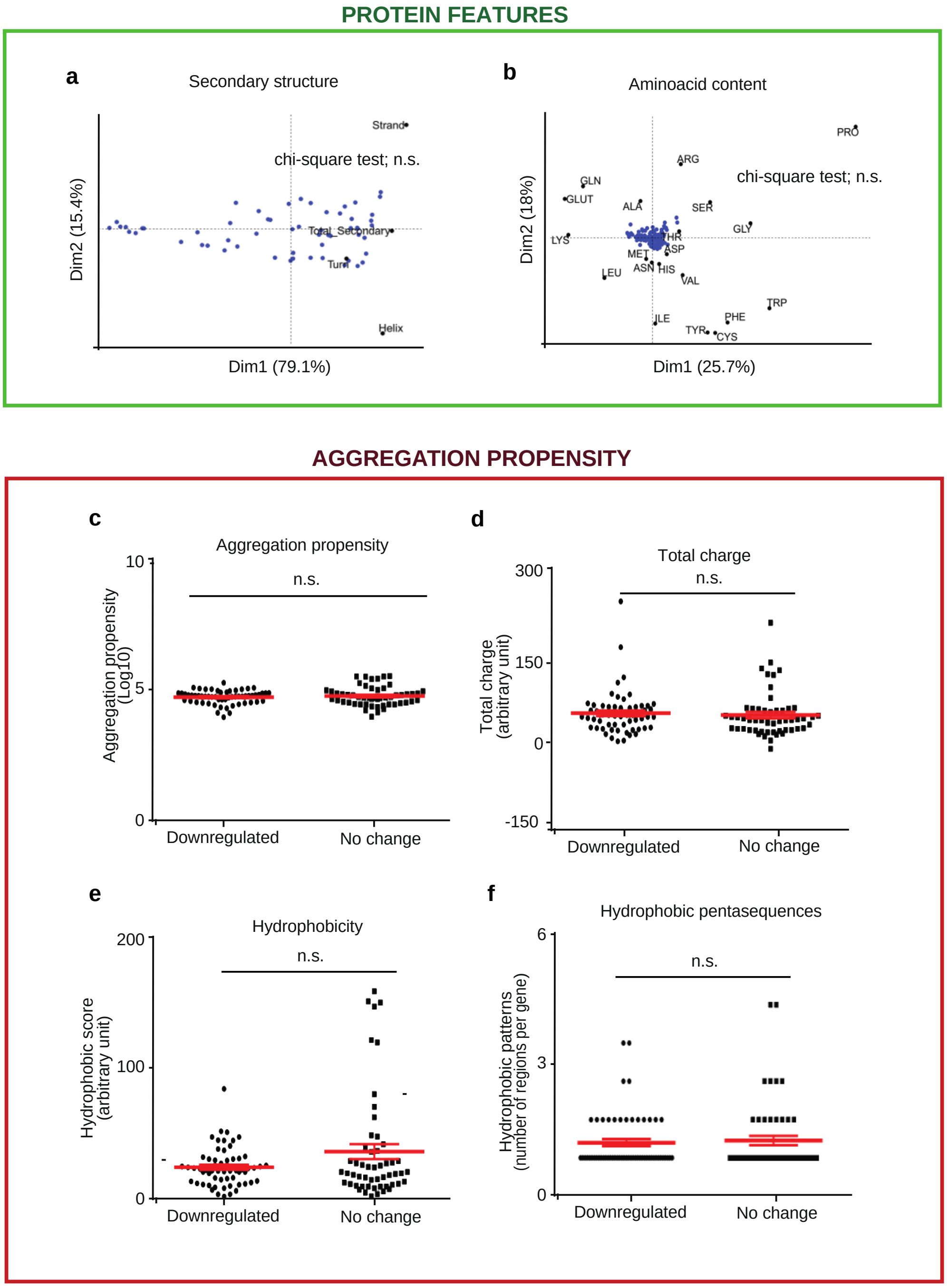

Supplementary Figure 5 related to Figure 4

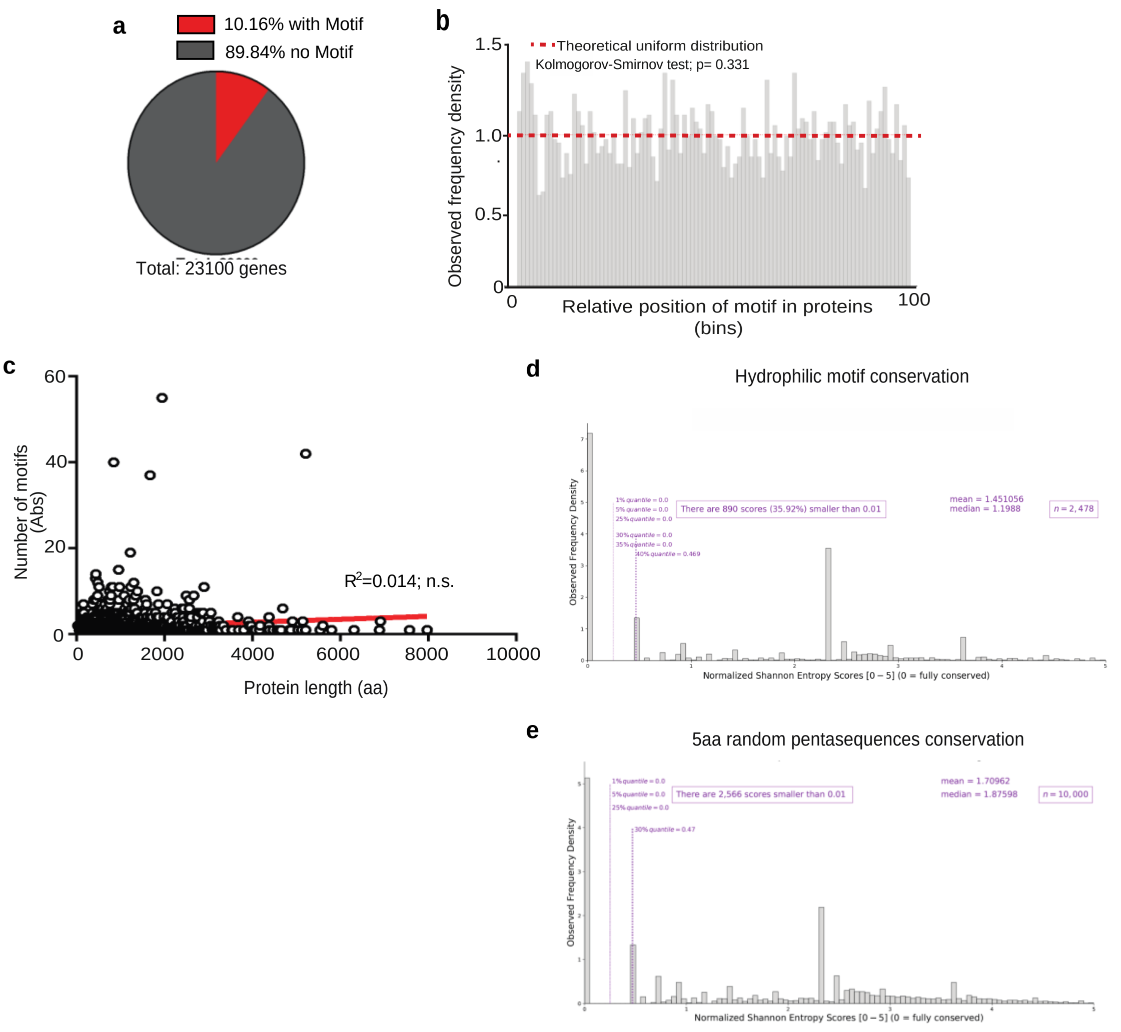

Supplementary Figure 6 related to Figure 5

103

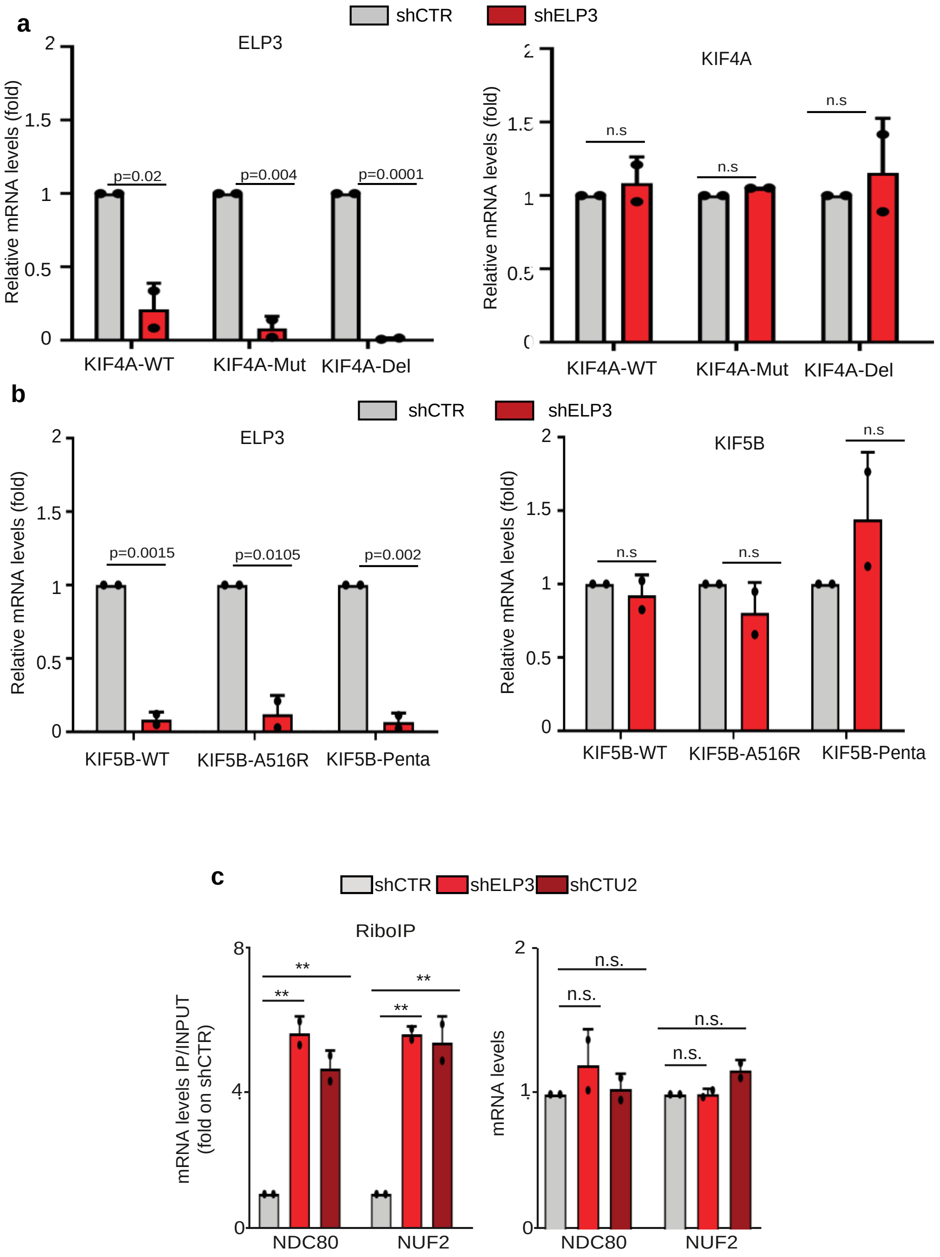

Supplementary Figure 7 related to Figure 5 and 6

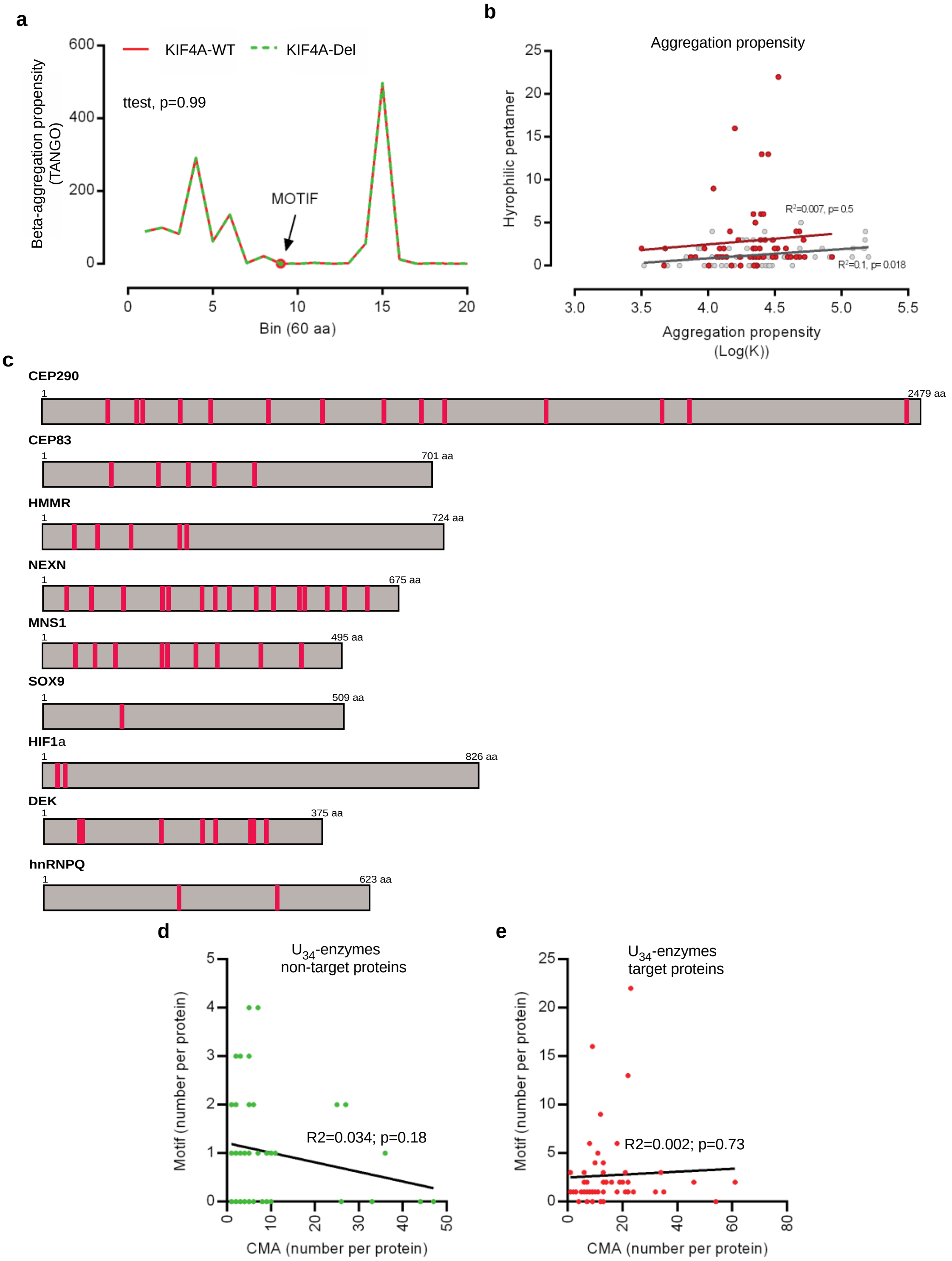

Supplementary Figure 8 related to Figure 7
