# Supplementary material for: Wobble tRNA modification and hydrophilic amino acid patterns dictate protein fate
Source: Nat Commun. 2021 Apr 15;12:2170. doi: 10.1038/s41467-021-22254-5 (PMC8050329; doi:10.1038/s41467-021-22254-5)
Supplement: Supplementary file 8 — Description of Additional Supplementary Files [file 41467_2021_22254_MOESM8_ESM.docx]

Description of additional supplementary information

Title: Supplementary dataset 1

Description: GO enrichment analysis of genes strongly enriched in all three U34-codons (AAA, GAA, CAA: q-value < 0.05)

Title: Supplementary Dataset 2

Description: sheet 1: enrichment in U34-codons in mRNAs encoding proteins down regulated upon ELP3 depletion.

sheet 2: RNA-seq data of BT549 cells depleted of ELP3 (enrichment in U34-codons)

sheet 3: enrichment in U34-codons in mRNAs encoding proteins of the kinesis family.

Title: Supplementary Dataset 3

Description: List of proteins whose expression is down regulated upon ELP3 depletion in BT549 cells.

Title: Supplementary Dataset 4

Description: Correlation analyses of the presence of the identified motif in different datasets

Title: Supplementary Dataset 5

Description: The antibodies, primers and shRNA sequences used in this study
