# Supplementary material for: Wobble tRNA modification and hydrophilic amino acid patterns dictate protein fate
Source: Nat Commun. 2021 Apr 15;12:2170. doi: 10.1038/s41467-021-22254-5 (PMC8050329; doi:10.1038/s41467-021-22254-5)
Supplement: Supplementary file 9 — Reporting Summary [file 41467_2021_22254_MOESM9_ESM.pdf]

nature research

Corresponding author(s): Pierre Close
Francesca Rapino

Last updated by author(s): 2020/11/18

Reporting Summary

Nature Research wishes to improve the reproducibility of the work that we publish. This form provides structure for consistency and transparency in reporting. For further information on Nature Research policies, see our Editorial Policies and the Editorial Policy Checklist.

Statistics

For all statistical analyses, confirm that the following items are present in the figure legend, table legend, main text, or Methods section.

n/a	Confirmed	
☐	☒	The exact sample size (*n*) for each experimental group/condition, given as a discrete number and unit of measurement
☐	☒	A statement on whether measurements were taken from distinct samples or whether the same sample was measured repeatedly
☐	☒	The statistical test(s) used AND whether they are one- or two-sided<br>*Only common tests should be described solely by name; describe more complex techniques in the Methods section.*
☐	☒	A description of all covariates tested
☐	☒	A description of any assumptions or corrections, such as tests of normality and adjustment for multiple comparisons
☐	☒	A full description of the statistical parameters including central tendency (e.g. means) or other basic estimates (e.g. regression coefficient) AND variation (e.g. standard deviation) or associated estimates of uncertainty (e.g. confidence intervals)
☐	☒	For null hypothesis testing, the test statistic (e.g. *F*, *t*, *r*) with confidence intervals, effect sizes, degrees of freedom and *P* value noted<br>*Give P values as exact values whenever suitable.*
☒	☐	For Bayesian analysis, information on the choice of priors and Markov chain Monte Carlo settings
☒	☐	For hierarchical and complex designs, identification of the appropriate level for tests and full reporting of outcomes
☐	☒	Estimates of effect sizes (e.g. Cohen's *d*, Pearson's *r*), indicating how they were calculated

Our web collection on statistics for biologists contains articles on many of the points above.

Software and code

Policy information about availability of computer code

Data collection	Proteomics: Swissprot H. sapiens protein database , GO_biological process database<br>Codon analysis: human genome database (hg38)
Data analysis	Proteomics: MaxQuant v1.6.2.3 (Max Planck Institute), Andromeda, Toppgene, Toppfun

For manuscripts utilizing custom algorithms or software that are central to the research but not yet described in published literature, software must be made available to editors and reviewers. We strongly encourage code deposition in a community repository (e.g. GitHub). See the Nature Research guidelines for submitting code & software for further information.

Data

Policy information about availability of data

All manuscripts must include a data availability statement. This statement should provide the following information, where applicable:

- Accession codes, unique identifiers, or web links for publicly available datasets
- A list of figures that have associated raw data
- A description of any restrictions on data availability

Proteomics data are available at ProteomeXchange Consortium (PXD019590 and PXD019620) and RNA-seq data are available at ArrayExpress (E-MTAB-9206). Open access databases used in this work include: Swissprot (H. sapiens protein database,https://www.uniprot.org/uniprot/?query=*&fil=organism%3A%22Homo+sapiens+%28Human%29+%5B9606%5D%22+AND+reviewed%3Ayes); Human genome database (https://www.ncbi.nlm.nih.gov/grc/human), GO_biological process database (http://geneontology.org/)

Field-specific reporting

Please select the one below that is the best fit for your research. If you are not sure, read the appropriate sections before making your selection.

☒ Life sciences ☐ Behavioural & social sciences ☐ Ecological, evolutionary & environmental sciences

For a reference copy of the document with all sections, see nature.com/documents/nr-reporting-summary-flat.pdf

Life sciences study design

All studies must disclose on these points even when the disclosure is negative.

Sample size	not applicable. The use of 2 or more replicates was chosen as a commontly accepted sample size for the different technologies.
Data exclusions	no data was excluded
Replication	All experiments were done in technical replicate, the number of biological replicates for each experiment is shown as single dots in the figures
Randomization	For the molecular biology experiments here performed no randomization was used as comontly accepted for these experimental designs.
Blinding	experiment were not blind

Reporting for specific materials, systems and methods

We require information from authors about some types of materials, experimental systems and methods used in many studies. Here, indicate whether each material, system or method listed is relevant to your study. If you are not sure if a list item applies to your research, read the appropriate section before selecting a response.

Materials & experimental systems

n/a	Involved in the study	
☐	☒	Antibodies
☐	☒	Eukaryotic cell lines
☒	☐	Palaeontology and archaeology
☒	☐	Animals and other organisms
☒	☐	Human research participants
☒	☐	Clinical data
☒	☐	Dual use research of concern

Methods

n/a	Involved in the study	
☒	☐	ChIP-seq
☐	☒	Flow cytometry
☒	☐	MRI-based neuroimaging

Antibodies

Antibodies used

All antibodies are listed in Table 5FLAG FLAG-M2 Mouse Sigma Life Science F1804
Alexa Flour 568 dye Alexa Flour 568 dye Mouse Thermo Fisher Scientific A11004

Name in the paper Antibody Spices Company Catalogue number
a-TUBULIN Alpha-Tubulin mono clone B-5-1-2 Mouse Sigma T6074
CELSR3 CELSR3/Flamigo Homolog 1 Rabbit Bethyl A305-188A-T
CENPE CENP-E Rabbit Bethyl A301-942A-T
CEP290 CEP290 Rabbit Bethyl A301-659A-T
CTU2 CTU2 Rabbit Abcam ab177160
ELP3 ELP3 Rabbit Cell Signaling 5728S
FLAG FLAG Rabbit Sigma Life Science F7425
GAPDH GAPDH Antibody (FL-335) Rabbit Santa Cruz sc-25778
HMMR CD168/RHAMM Rabbit Bethyl A304-783A-T
HSP90 Hsp90 alpha/beta (H-114) Rabbit Santa-Cruz sc-7947
KIF14 KIF14 Rabbit Bethyl A300-233A-T
KIF15 KIF15 Rabbit Bethyl A302-706A-T
KIF4A KIF4A Rabbit Bethyl A301-073A-T
KIF5B KIF5B Rabbit Bethyl A304-306A-T
KIF7 KIF7 Rabbit Bethyl A302-709A-T
MNS1 MNS1 Rabbit Abcam ab99146
NEXN Nexilin Rabbit Bethyl A303-806A-T
p-H3 phospho Histone 3 Rabbit Santa-Cruz sc-8656-R

Validation	Validation was performed from the manufacturer. Exemple of western blots and/or Immunohistochemestry on human cell lines (fibrobast or Hela cells) are present in the respective datasheets.

Eukaryotic cell lines

Policy information about cell lines

Cell line source(s)	BT549 (ATCC® HTB-122)<br>MDA-MB231 (ATCC® HTB-26)<br>MCF7 (ATCC® HTB-22)
Authentication	none of the cell lines used were authenticated
Mycoplasma contamination	Cell lines are tested routinely for mycoplasma. Only cells negative to mycoplasma contamination were used.
Commonly misidentified lines (See ICLAC register)	No commontly misidentified lines were used.

Flow Cytometry

Plots

Confirm that:

☒ The axis labels state the marker and fluorochrome used (e.g. CD4-FITC).

☒ The axis scales are clearly visible. Include numbers along axes only for bottom left plot of group (a 'group' is an analysis of identical markers).

☒ All plots are contour plots with outliers or pseudocolor plots.

☒ A numerical value for number of cells or percentage (with statistics) is provided.

Methodology

Sample preparation	Protein aggregates formation was monitored by FACS using PROTEOSTAT® Aggresome Detection Kit (ENZO) according to manufacturer instructions.
Instrument	BD FACSCanto™ II Flow Cytometry System
Software	FlowJo
Cell population abundance	A minimum of 5000 events was used for all FACS measurements
Gating strategy	No gating was performed. Aggresome propensity factor (APF) was calculated using the formula: 100*(mean fluorescence intensity depleted - mean fluorescence intensity control) / mean fluorescence intensity depleted.

☒ Tick this box to confirm that a figure exemplifying the gating strategy is provided in the Supplementary Information.
