# Supplementary material for: Wobble tRNA modification and hydrophilic amino acid patterns dictate protein fate
Source: Nat Commun. 2021 Apr 15;12:2170. doi: 10.1038/s41467-021-22254-5 (PMC8050329; doi:10.1038/s41467-021-22254-5)

Main figures

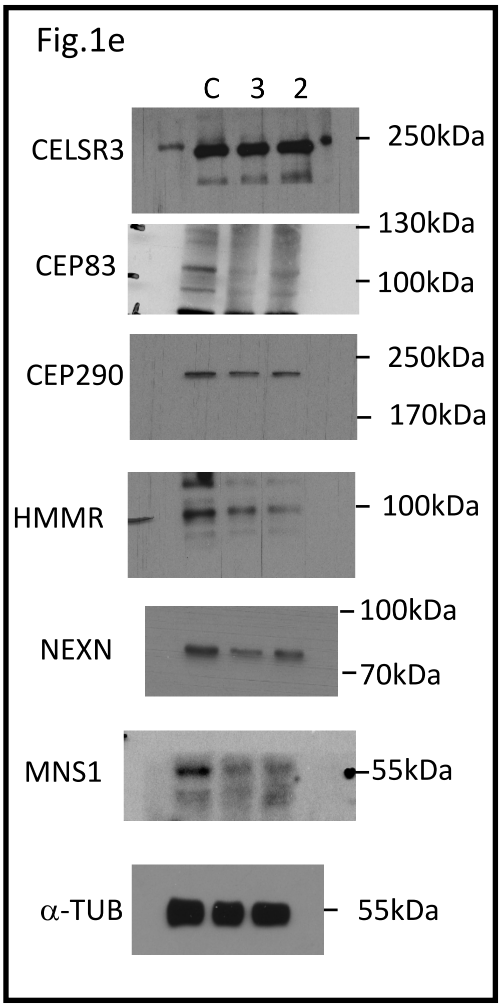

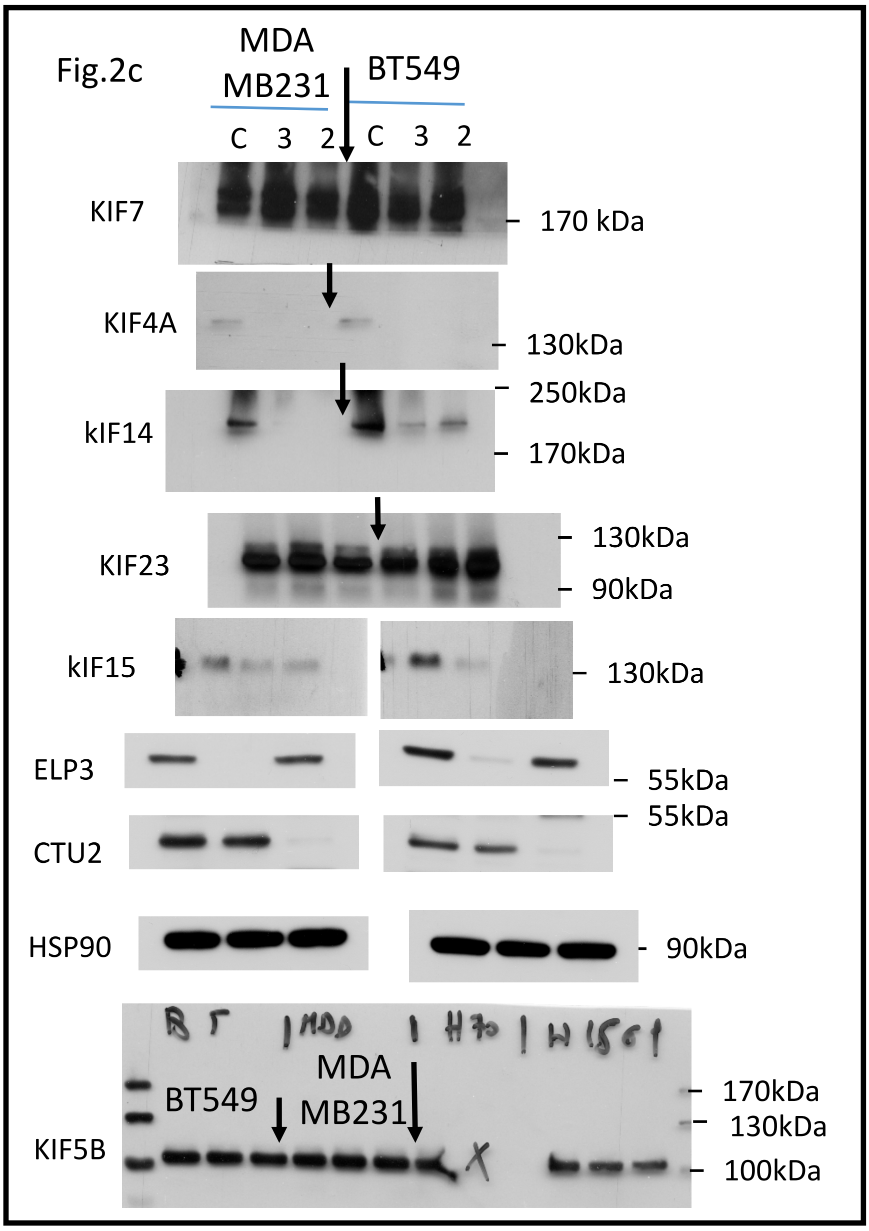

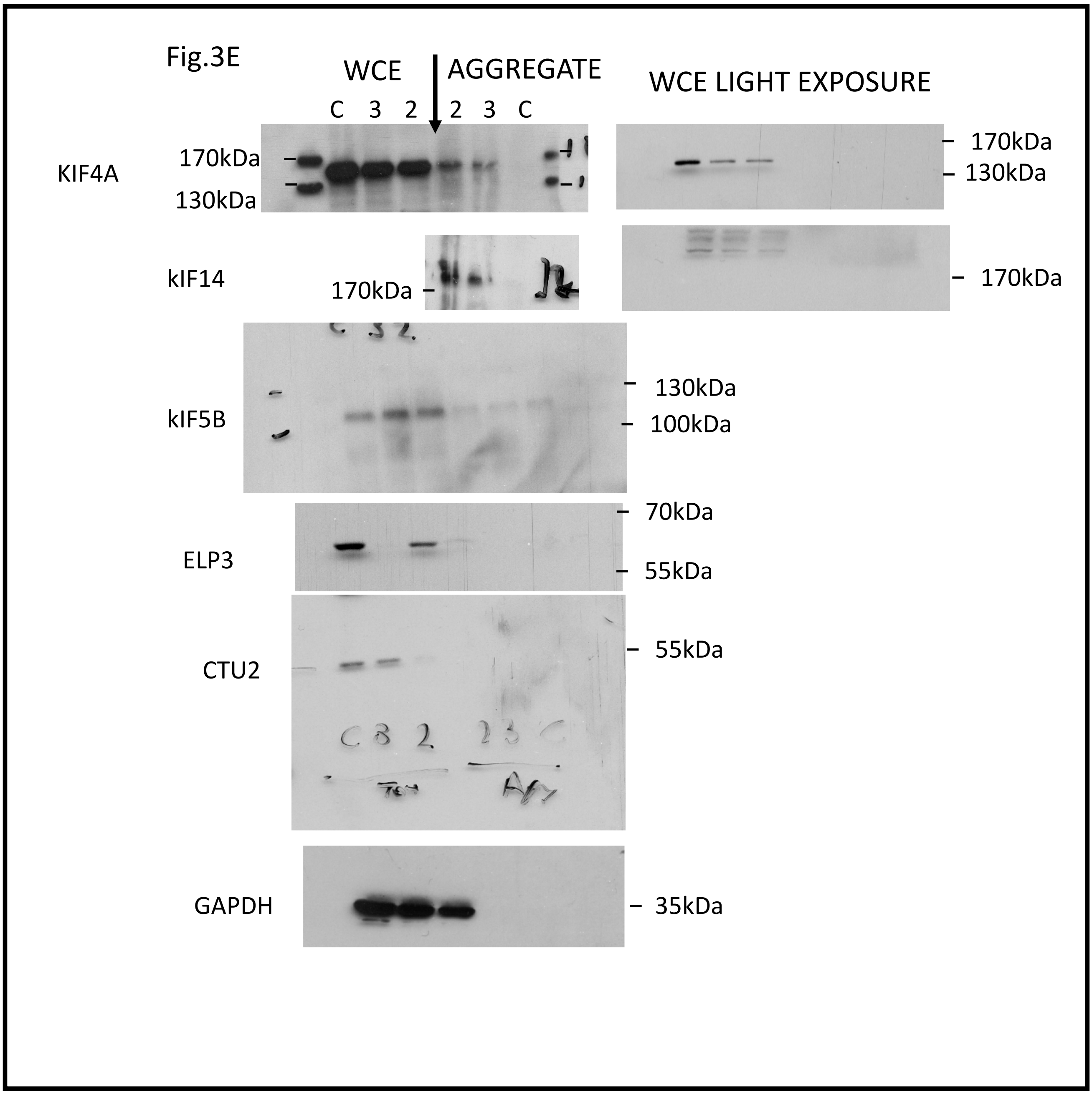

Fig. 3G

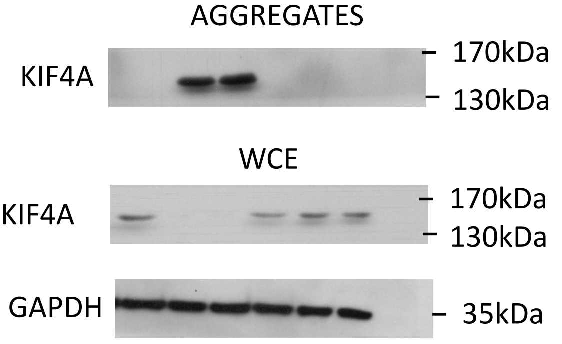

Fig.5D

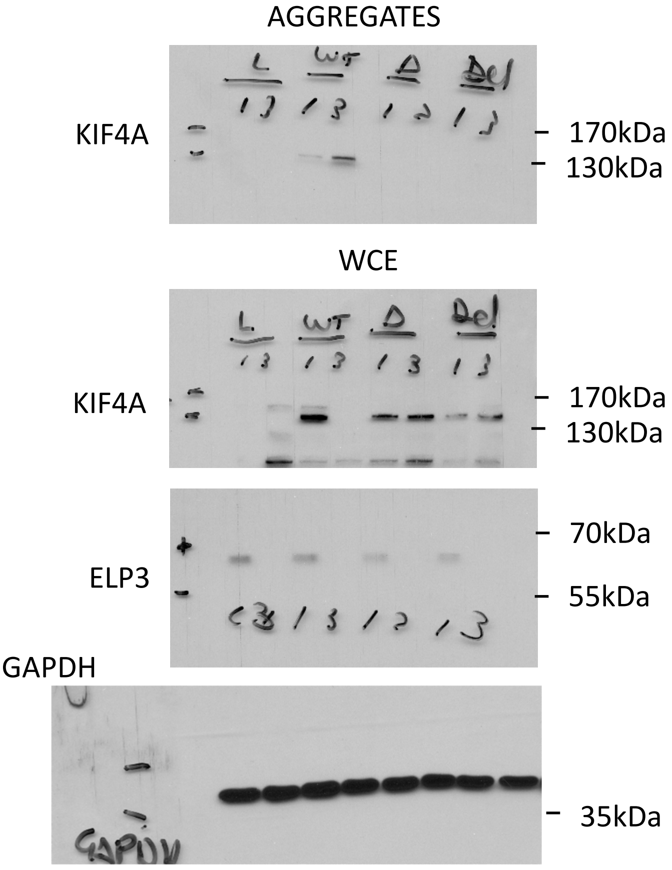

Fig.5F

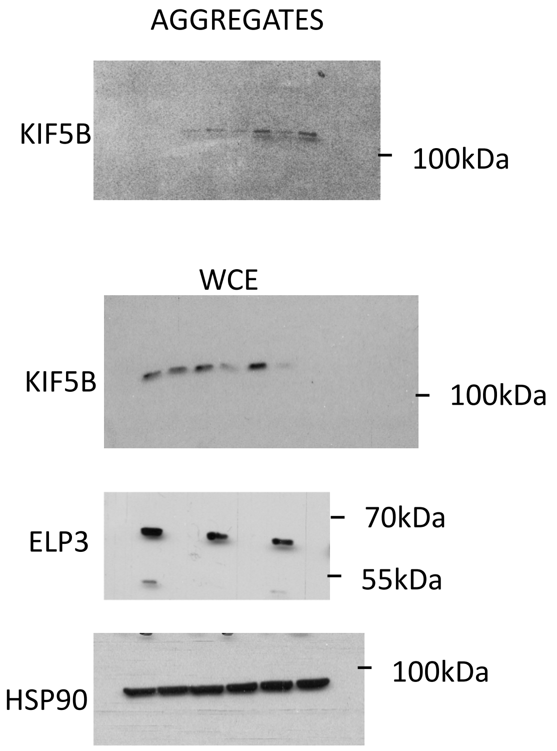

Fig.6B

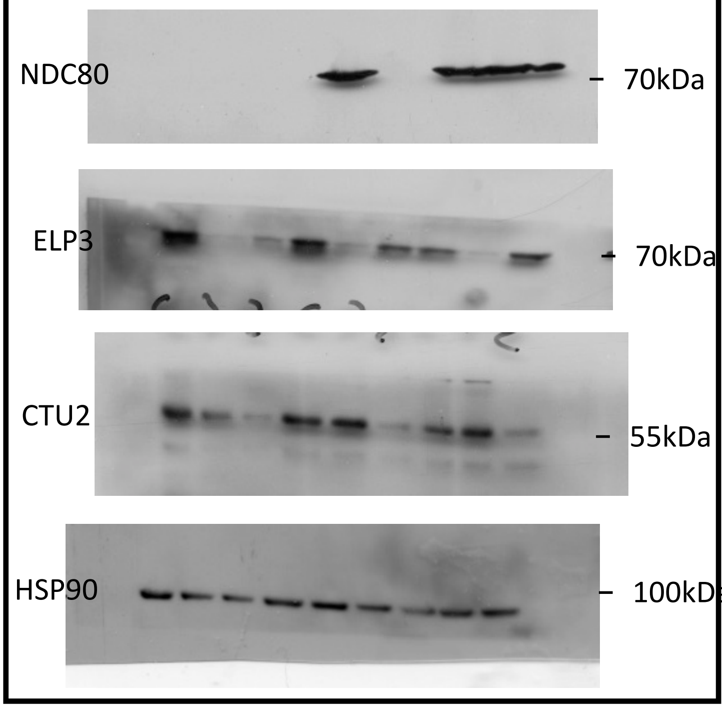

Fig.6C

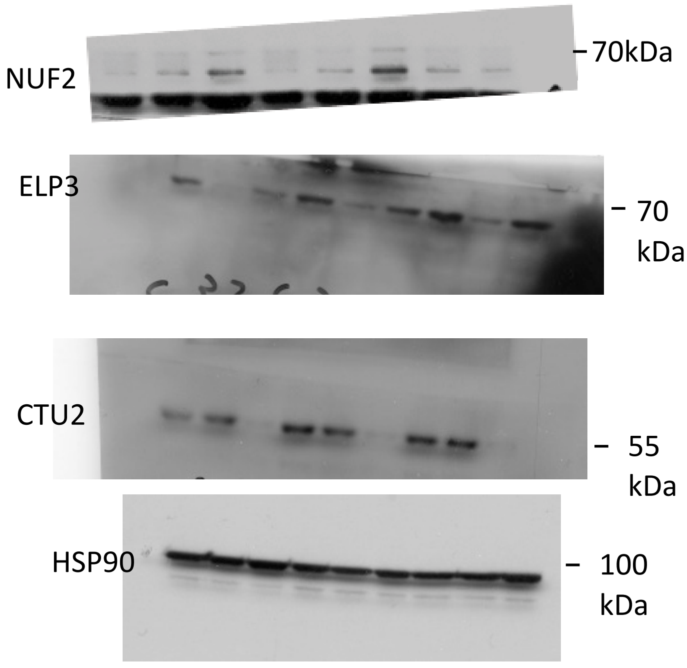

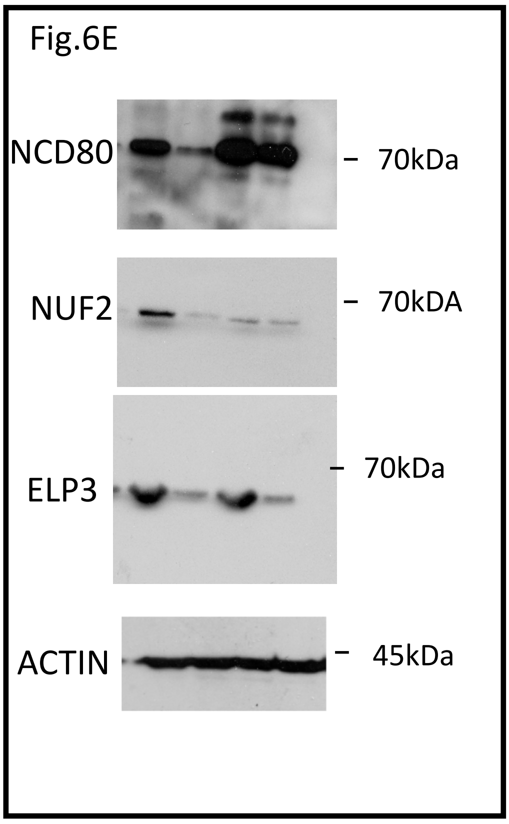

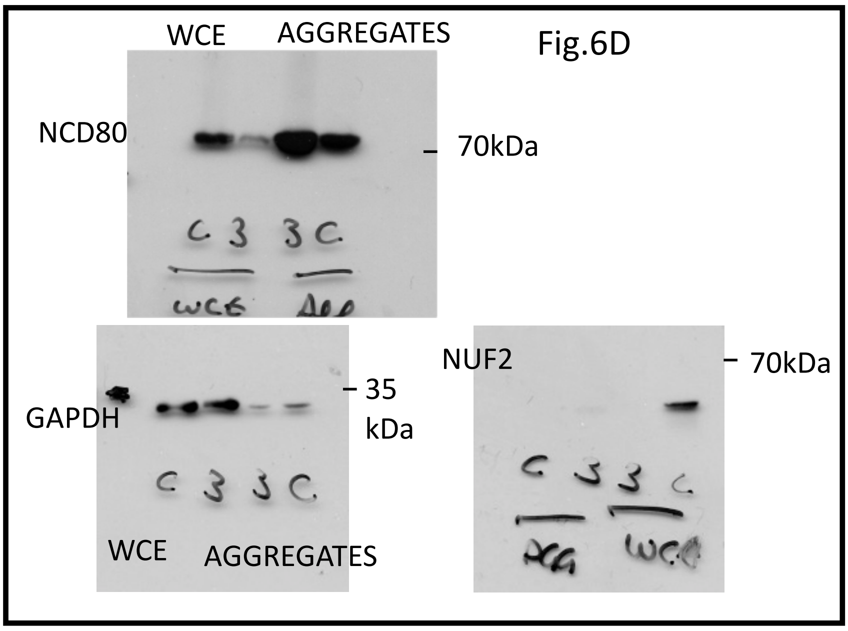

Supplementary figures

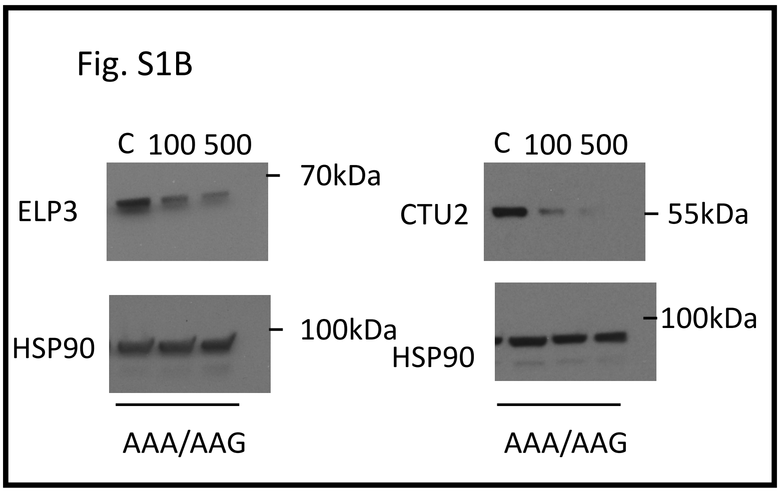

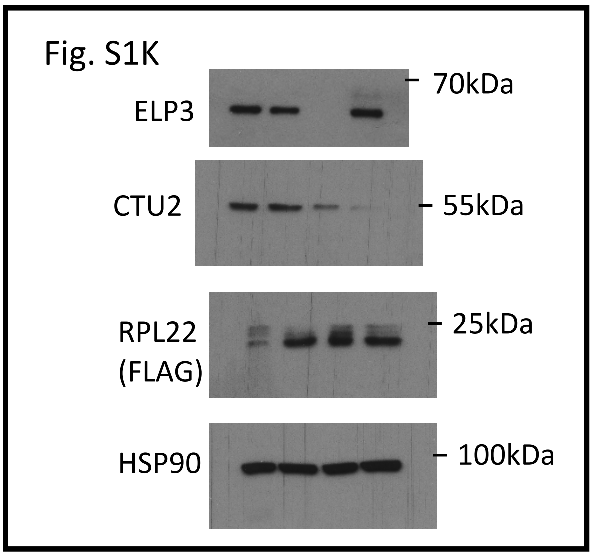

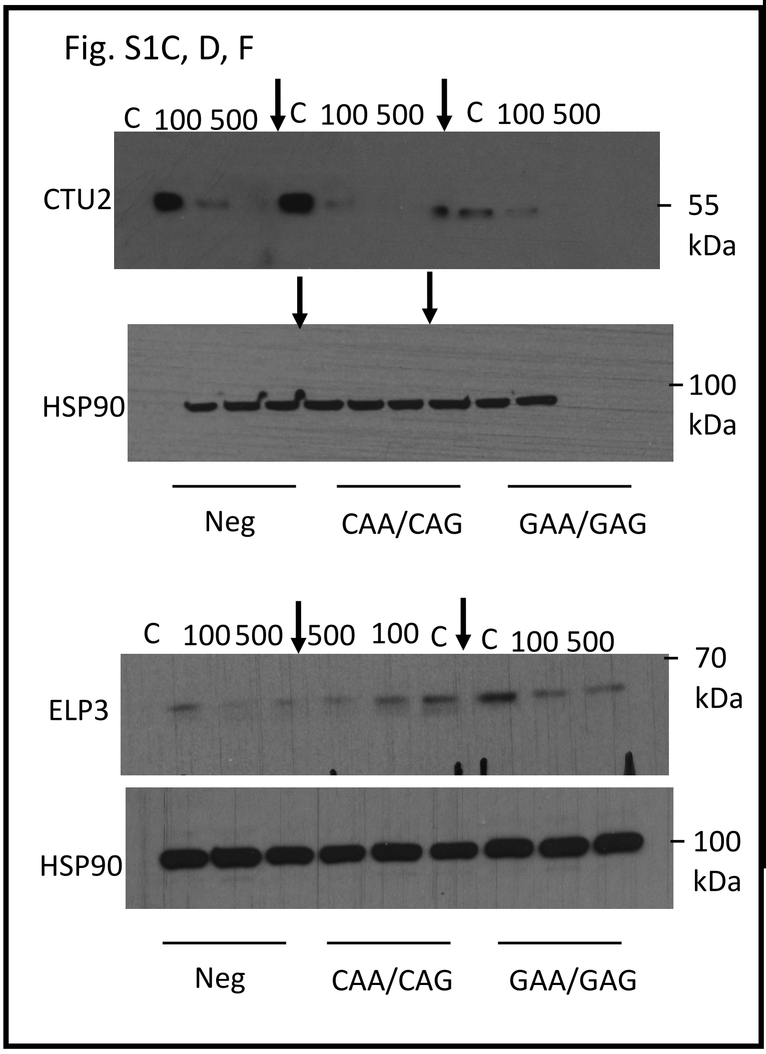

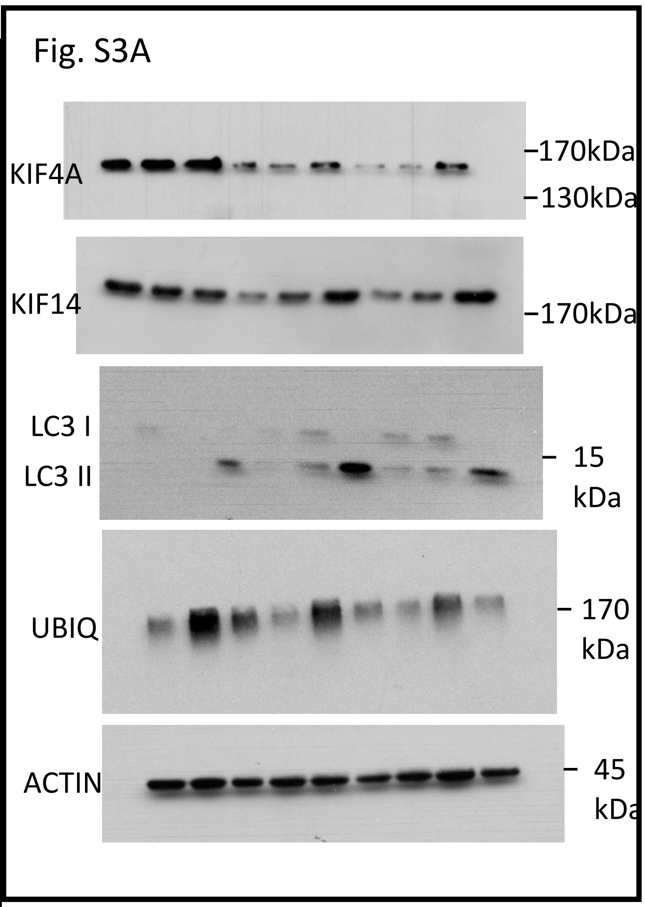

Gating strategy (Fig 3 a-c; Suppl. Fig 3c)

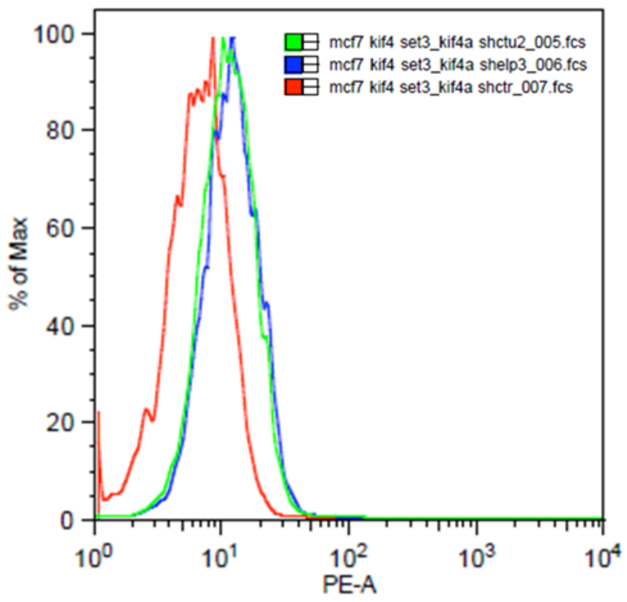

Supplement: Supplementary file 10 — Source Data [file 41467_2021_22254_MOESM10_ESM.pdf]
